# Purification of Wet-Process Phosphoric Acid via Donnan Dialysis with a Perfluorinated Sulfonic Acid Cation-Exchange Membrane

**DOI:** 10.3390/membranes11040298

**Published:** 2021-04-20

**Authors:** Qin Zhong, Tao Luo, Zhengjuan Yan, Lin Yang, Zhiye Zhang, Xinlong Wang

**Affiliations:** Ministry of Education’s Research Centre for Comprehensive Utilization and Clean Process Engineering of Phosphorus Resources, School of Chemical Engineering, Sichuan University, Chengdu 610065, China; m17854237480@163.com (Q.Z.); tao.luo@scu.edu.cn (T.L.); yanglyangl@163.com (L.Y.); zhiyezhang@scu.edu.cn (Z.Z.); wangxl@scu.edu.cn (X.W.)

**Keywords:** wet-process phosphoric acid, Donnan dialysis, perfluorinated sulfonic acid cation-exchange membrane, acid purification, electromembrane process

## Abstract

This work reports the application of an electromembrane process, Donnan dialysis (DD), for the purification of so-called wet-process phosphoric acid (WPA). Nitric acid is used as the stripping solution to remove metallic cations (mostly Fe^3+^, Al^3+^, and Mg^2+^) that are harmful to the further processing of WPA. The paper first presents a set of experimental data on the measurements of the metallic cation fluxes through a perfluorinated sulfonic acid cation-exchange membrane. Not only WPA, but also synthetic phosphoric acid solutions with mixed metallic cations (MPA) and with a single metallic cation (SPA) were studied. This confrontation confirms (1) that the order of metallic cations fluxes is Mg^2+^ > Al^3+^ > Fe^3+^; (2) that, compared with MPA, the purification effect of WPA causes only negligible change; (3) that, by comparing the DD processes with SPA and MPA solutions, the reason for the low transmembrane fluxes of Fe^3+^ and Al^3+^ could be explained by the large ionic charge and large hydrated ion radius. Furthermore, by analyzing the ion composition of membranes equilibrated in SPA solutions, we conclude that the forms of cations in the membrane are most likely Fe^3+^, Al^3+^, and Mg^2+^.

## 1. Introduction

Ion-exchange membranes (IEMs) are an important class of dense polymeric membranes that bear fixed charges and mobile counterions in the polymer matrix, in the presence of polar solvents (e.g., water). These membranes can selectively allow the passage of oppositely charged ions (counterions), while obstructing similarly charged ions (coions) [1,2]. Commonly used IEMs are anion-exchange membranes and cation-exchange membranes (CEMs). Due to the nice selectivity of IEMs and their simple operation, they are used in various electromembrane processes, such as Donnan dialysis (DD), electrolysis, (reverse) electrodialysis, and diffusion dialysis.

As a membrane-based equilibrium process, DD is employed as a useful separation technology for chemical analysis, water treatment [3,4], and drug separation or purification, and especially for the separation and recovery of precious metals [2]. In the field of wet-process phosphoric acid (WPA) purification, metallic cations also need to be removed because these metallic cations not only increase the density of phosphoric acid, but also easily lead to the formation of insoluble phosphates, especially for iron and aluminum ions that seriously affect the downstream processing of WPA [5]. However, due to the complex composition of WPA, harsh operation conditions, and severe scaling problems, the purification of WPA has been a challenging topic.

Until now, the purification of WPA in industry relied mainly on solvent extraction or chemical precipitation. The solvent extraction method has advantages like a relatively high extraction ratio of phosphoric acid, good selectivity, easy phase separation, good flow properties of streams, and it is cheap and easy to realize. Both academia and industry have done a lot of work on extractant screening and purification performance evaluation. However, the extraction method uses a large amount of organic extractants (e.g., dibutyl sulfoxide [6] and cyclohexanol [7]), and the process produces poorly water-soluble sludge acid. Alternatively, the chemical precipitation method uses chemical precipitation agents, such as ammonia [5], NaOH [8], ammonium nitrate with hydrofluoric acid [9], and lime milk to precipitate the metallic cations in WPA in the form of phosphoric acid (double) salts. This process introduces chemical reagents into the produced phosphoric acid and causes the waste of phosphorus resources. Besides, this process is lengthy. In short, both purification methods have their limitations.

There are some reports on the application of ion exchange or membrane technology in the purification of phosphoric acid. In terms of ion exchange, Tang et al. used ion-exchange resins to remove the aluminum cations from synthetic phosphoric acid solutions [10]. Amin et al. also investigated the removal of iron from WPA by Puromet MTS9570 resin [11]. However, the disadvantage of ion exchange is that the solid-to-liquid ratio used is large, and the resin is loose and porous, which will cause excessive entrainment. There are also some studies on promising membrane processes for the removal of target metallic cations. Sonoc et al. utilized Donnan dialysis to recover lithium, nickel, cobalt, and manganese from lithium-ion batteries [12]. Diallo et al. used MPF34 nanofiltration membranes to study the migration of iron, the main metal contaminant, in WPA [13]. Therefore, it is, in principle feasible to choose a suitable ion-exchange membrane to purify WPA via the DD process.

In this study, DD was applied to purify a typical WPA. Synthetic phosphoric acid solutions with the same metallic cation composition as WPA (MPA), each with only one type of cation as the corresponding component in WPA (SPA), were employed to study the removal ratios of different metallic cations in the DD process. The existing form of cations in the membranes was also studied by comparing the molar ratios of metallic cations and phosphorus in the membrane.

## 2. Materials and Methods

### 2.1. Chemicals and Membranes

The WPA solution came from a phosphate ore processing plant located in Inner Mongolia in China. The characteristic contents of each component in this WPA are shown in Table 1. To further understand the ion-exchange process of the Fe, Al, and Mg species in the purification process, three different kinds of synthetic phosphoric acid solutions with each single metallic cation (SPA) of the same concentration as in WPA were prepared.

A commercial phosphoric acid solution with a H_3_PO_4_ mass fraction of 85% was used in the preparation of synthetic phosphoric acid solutions. The NaCl, MgO, and Al_2_(SO_4_)_3_ used were from Chengdu Kelong Chemical Co., Ltd. Fe_2_(SO_4_)_3_ was purchased from Chengdu Jinshan Chemical Reagent Co., Ltd. (China). All chemicals were of analytical pure grade. MPA was prepared according to the contents of target components listed in Table 1.

A perfluorinated sulfonic acid cation-exchange membrane (CEM) was used in this study. Table 2 lists the properties of this CEM. This CEM (DMR100M) was supplied by the Shandong Dongyue Future Hydrogen Energy Material Co., Ltd. (China).

### 2.2. Analysis Methods

An inductively coupled plasma optical emission spectrometer (ICP-OES, Optima 7000DV, Platinum Elmer, Waltham, MA, USA) and X-ray fluorescence spectroscopy (XRF, S8, Tiger, Bruker, Karlsruhe, Germany) were used to detect the contents of metallic cations in solutions. Fluorine and phosphorus contents in solutions were detected with an ion meter (PXS-270, Shanghai INESA Scientific Instrument, Shanghai, China) and UV spectrophotometer (UV-3600, Shimadzu, Kyoto, Japan), respectively. A scanning electron microscope (SEM, Vega 3 SBH, Tescan, Brno, Czech Republic) was used to observe the morphology of the membrane.

### 2.3. Donnan Dialysis Process

**Scheme 1:**
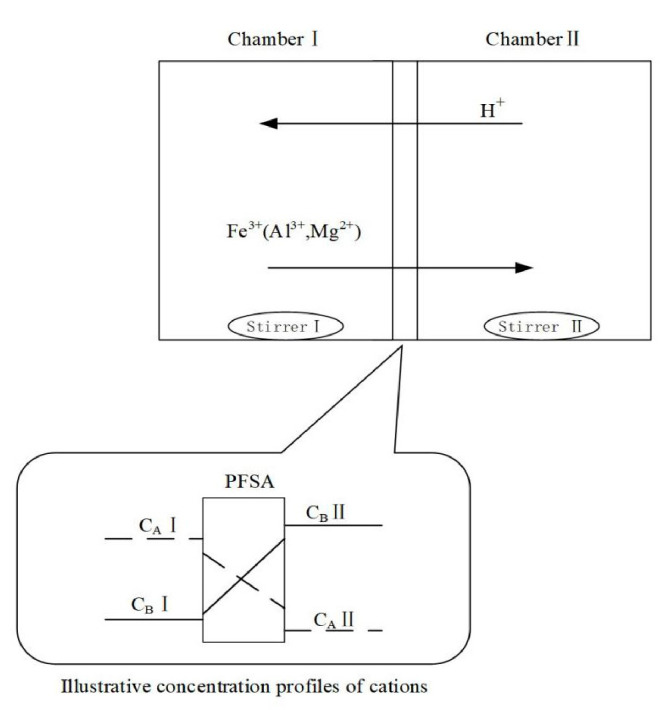
Schematic diagram of the experimental Donnan dialysis process for the purification of WPA. PFSA is short for perfluorinated sulfonic acid.

The experiment of using the DD process for WPA purification is schematically shown in Scheme 1. Fe^3+^ (Mg^2+^ and Al^3+^) is the target impurity cation and the driving ion is H^+^ in this case. The membrane area, the concentration of stripping (nitric acid solution), and the starting solution volumes of both the stripping solution and the WPA feed are 1.77 cm^2^, 5 mol L^−1^, and 9 mL, respectively. The phosphoric acid and nitric acid solutions were well mixed in Chambers I and II via magnetic stirrers, respectively. Intensive agitation was assured to diminish the resistance of the liquid films on both sides of the membrane surfaces. The perfluorinated sulfonic acid CEM was clamped between the two chambers of the cell. In this circumstance, the metallic cations in the phosphoric acid solution could be driven against their concentration gradients, as Moya described [14], and did exhibit uphill transport across the membrane. This uphill transport of metallic cations was caused by the proton concentration difference between Chambers I and II. The ratio of removal (towards metallic cations) was chosen to express the extent of purification, and it was calculated via the following equation:

(1) $$\mathrm{Ratio}\;\mathrm{of}\;\mathrm{removal}=\frac{C_{t,\mathrm{II}}*V_{\mathrm{II}}}{C_{0,I}*V_{I}}*100\%$$

Among them, C_0,I_ and C_t,II_ are the concentrations of cations at time zero in Chamber I and at time t in Chamber II, respectively; V_I_ and V_II_ are the volumes of electrolyte solutions in Chambers I and II, respectively.

### 2.4. Absorption Experiments

A certain amount of the CEM was pretreated and then soaked in SPA for 48 h to ensure complete ion exchange between the membrane and the synthetic solutions; then the CEM was washed with deionized water to remove the absorbed free salts and acids. Afterwards, the CEM was soaked in 50 mL of 1 mol L^−1^ NaCl solution for 48 h to allow sodium ions to replace the metal ions in the CEM. Lastly, the phosphorus content and the metal ion content in the desorbing solutions were measured by UV spectrophotometer and ICP-OES, respectively. For Fe^3+^-SPA, Al^3+^-SPA, and Mg^2+^-SPA, the membrane areas used in the absorption experiments were 83, 85, and 83 cm^2^, respectively.

## 3. Results and Discussions

### 3.1. Membrane Morphology Analysis

**Figure 1:**
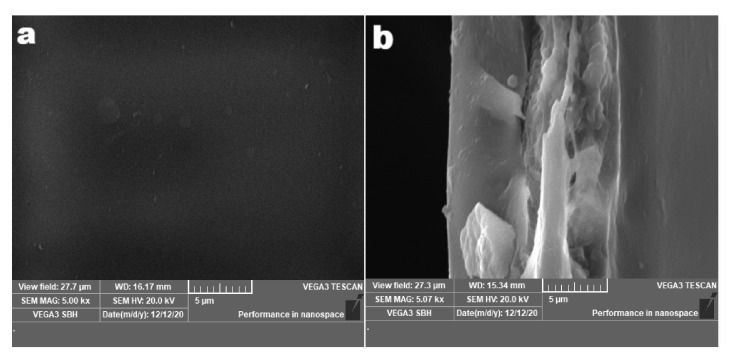
Scanning electron micrograph (SEM) picture of (**a**) the surface of the perfluorinated sulfonic acid cation-exchange membrane (CEM) DMR100M and (**b**) the cross-section of the membrane.

Figure 1 shows the SEM graphs of the perfluorinated sulfonic acid CEM DMR100M. As shown in Figure 1a, there are no observable holes or defects on the surface of the membrane, confirming that the membrane surface is dense at dry state. It can be seen from the cross-sectional graph of the membrane in Figure 1b that the membrane cross-section does not have any finger-like pores or sponge structures as observed in porous membranes. In conclusion, this perfluorinated sulfonic acid CEM is a dense film at dry state.

### 3.2. Removal of Metallic Cations from Industrial Wet-Process Phosphoric Acid

**Figure 2:**
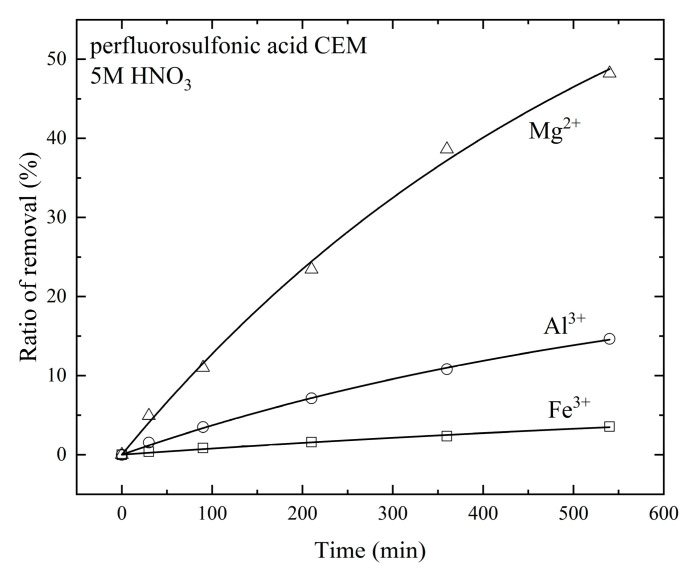
Removal ratios of different metallic cations from WPA as a function of time.

Figure 2 shows the relationship between the removal ratios of different metallic cations from WPA and time in the DD process with nitric acid as the stripping solution. It is obvious that the order of removal ratios of metallic cations is Mg^2+^ > Al^3+^ > Fe^3+^. The removal ratio of Mg^2+^ is much greater than those of the other two metal ions. After 9 h, the removal ratio of Mg^2+^ reached 48.23%. At the same time, the removal ratios were only 14.64% and 3.35% for Al^3+^ and Fe^3+^, respectively; the accuracy in determining the ratios presented is 6%. This experimental result is in line with expectations. In DD, the exchange of ions is a chemical reaction, and ions react with the sulfonic acid groups on and in the CEM to replace protons or other metal ions that had previously been there. The number of such groups in the membrane is certain. The greater number of charges carried by the ions, the more fixed groups are involved, and the mass transfer ratio of the moving ion is correspondingly smaller. This is the main reason for the biggest mass transfer ratio, seen in Mg^2+^, which has a lower charge. Meanwhile, the hydrated radius of ions is also an important factor for explaining the mass transfer ratio. Although the CEM is treated as a homogeneous membrane at a macroscopic scale, it contains some “pores” filled with water for ions to pass through. The larger the hydrated radius of ions is, the more difficult it is for them to pass through the membrane. The charge numbers of Fe^3+^ and Al^3+^ are the same, but the hydrated radius of Fe^3+^ is larger than that of Al^3+^ [1], which means that the mass transfer ratio of Fe^3+^ in the DD process will be smaller than Al^3+^.

### 3.3. Removal of Metallic Cations from Synthetic Mixed Metallic Cations Phosphoric Acid

**Figure 3:**
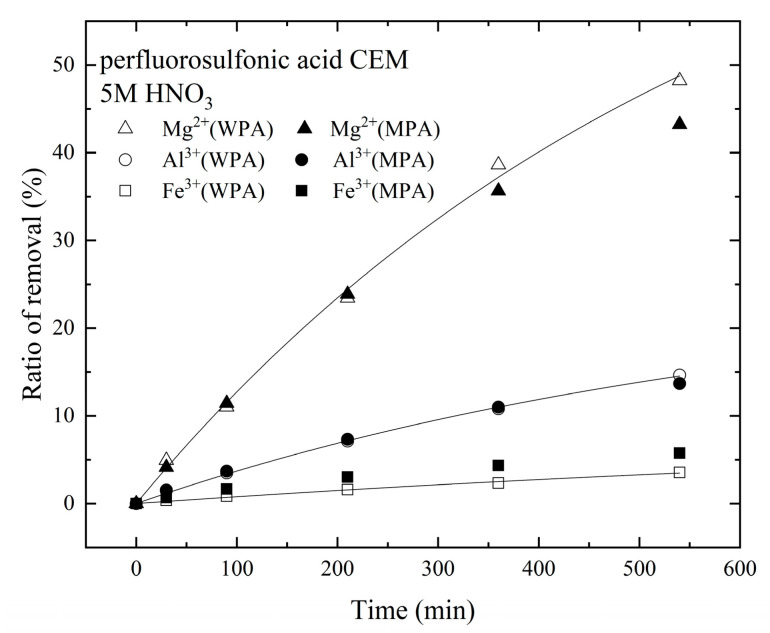
Removal ratios of metallic cations (filled symbols) as a function of time, from MPA that is a synthetic phosphoric acid solution with the same cation compositions as WPA. The removal ratios for WPA (hollow symbols) are plotted as references.

In order to investigate the mutual influence of impurity cations in phosphoric acid on metal removal in the DD process, MPA was used. The removal ratio data are shown in Figure 3. The removal ratios of MPA and WPA are very close, and the slight difference may come from the influence of other impurity ions (trace K^+^, Na^+^, and some Ca^2+^ ions) in WPA. Although most Al^3+^ will form stable AlF_6_^3−^ with F^−^, the remaining Al^3+^ will form AlF^2+^ or AlF_2_^+^ with F^−^ [15], which leads to the reduction of the charge number of the complex ions and the increase in the flux of Al^3+^.

### 3.4. Removal of Metallic Cations from Synthetic Single Metallic Cations Phosphoric Acid

**Figure 4:**
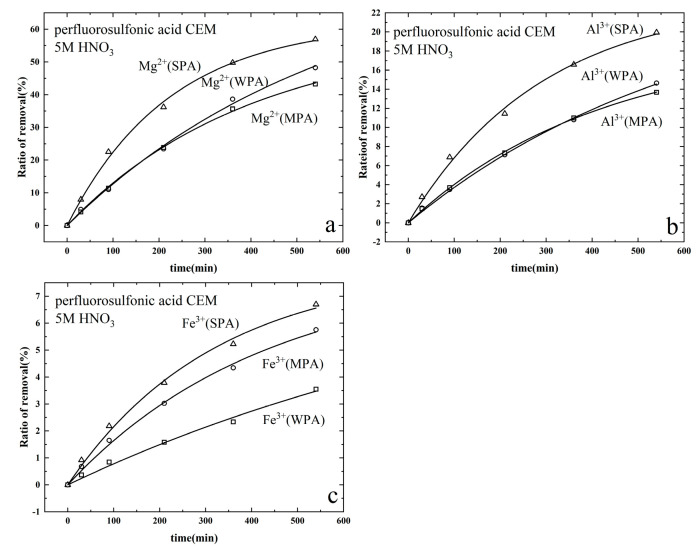
Removal ratio of (**a**) Mg^2+^, (**b**) Al^3+^, and (**c**) Fe^3+^ as a function of time, from SPAs that are synthetic phosphoric acid solutions, which each only contain one type of cation, as corresponding content of the same cation in WPA. The removal ratios from WPA and MPA are plotted as references.

In this case, the feed is SPA and the other experimental conditions are kept unchanged. The experimental data of the removal ratios of three metallic cations are summarized, and the results are shown in Figure 4. For the Mg^2+^, Al^3+^, and Fe^3+^-SPA, the average mass transfer rates in the first 30 min were 0.0261, 0.0062, and 0.0026 mol m^−^^2^ min^−^^1^. The removal ratio of Mg^2+^ can be improved by 13% compared with that in the MPA, but the removal ratio of ${\mathrm{Al}}^{3+}$ is only improved by 6.27%, possibly due to the slow mass transfer rate of Al^3+^ ions in the membrane. The removal ratio of ${\mathrm{Fe}}^{3+}$ is improved by less than 1%. This shows that the mass transfer resistance of ${\mathrm{Fe}}^{3+}$ and ${\mathrm{Al}}^{3+}$ in the DD process is much greater than that of ${\mathrm{Mg}}^{2+}$. The result is consistent with the removal ratio order of metallic cations.

According to the comparison of the absolute removal amount of metallic cations in different experiments, as summarized in Table 3, the total removal amount of charges carried by the removed metallic cations in WPA is the largest, reaching 2.48 mequ. Secondly, the charge amount carried by removed metallic cations in MPA acid is 2.30 mequ. For the ${\mathrm{Mg}}^{2+}$, ${\mathrm{Al}}^{3+}$, ${\mathrm{Fe}}^{3+}$ and -SPA, the charge amounts removed are 1.98, 0.72, and 0.30 mequ., respectively. For all experiments, the driving force for the impurity cation removal (proton concentration difference) was large enough, and the difference in the transfer amount of charges carried by removed cations may come from the different diffusion rates of cations in the membrane, instead of the metallic cations concentration in the feed. Base on this result, we can conclude that the order of metallic cation fluxes is Mg^2+^ > Al^3+^ > Fe^3+^.

### 3.5. Existing form of Metallic Cations in the Membrane

The basic formula for the quantitative treatment of ion transfer across the CEM, i.e., the Nernst–Planck equation, requires knowledge of the existing form of the metallic cations in the membrane. Equation (2) below describes the target ion flux in the DD process, with J_A_, D_A_, q_A_, and Z_A_ being the flux, self-diffusion coefficient, concentration in the membrane, and charge of Ion A, respectively.

(2) $$J_{A}=-D_{A}D_{B}\frac{Z_{A}^{2}q_{A}+Z_{B}^{2}q_{B}}{Z_{A}^{2}D_{A}q_{A}+Z_{B}^{2}D_{B}q_{A}}\frac{{\mathrm{dq}}_{A}}{\mathrm{dx}}$$

Obviously, the charge of the ion has a large effect on the mass transfer rate in the mass transfer process in the membrane. In order to determine in which forms these metallic cations exist in the CEM, absorption experiments have been done, and the data are shown in Table 4.

According to the molar ratio of a metal ion and the corresponding phosphorus content in the same membrane specimen, it can be concluded that most of the iron, aluminum, and magnesium ions in the CEM are present in the form of Fe^3+^, Al^3+^, and Mg^2+^, respectively.

## 4. Conclusions

In this work, a Donnan dialysis process with a perfluorinated sulfonic acid cation-exchange membrane (CEM) as the separator, and nitric acid as the stripping solution, is proposed to remove metallic cations (Mg^2+^, Al^3+^, and Fe^3+^) from wet-process phosphoric acid (WPA) solutions. The possibility of employing the Donnan dialysis to purify WPA is verified. Based on the ion composition of membranes equilibrated with synthetic phosphoric acid solutions each with a single metallic cation, it is concluded that the existing forms of the metallic cations in the CEM are Mg^2+^, Al^3+^, and Fe^3+^. This work would be of considerable relevance for the application of the electromembrane processes toward the purification of WPA.

## Figures and Tables

**Table 1:** Characteristic composition of the wet-process phosphoric acid (WPA) solution employed.

**Target Components**	**P**_**2**_ **O**_**5**_ **(H**_**3**_ **PO**_**4**_ **)** **[wt.%]**	**Fe** ^**3+**^ **[wt.%]**	**Al** ^**3+**^ **[wt.%]**	**Mg** ^**2+**^ **[wt.%]**
	26.57 (36.67)	0.66	0.27	0.39
Other components	**Ca** ^**2+**^ **[wt.%]**	**K** ^**+**^ **[wt.%]**	**Na** ^**+**^ **[wt.%]**	**F** ^**−**^ **[wt.%]**
	0.08	0.03	0.01	1.47

**Table 2:** Characteristics of the perfluorinated cation-exchange membrane used in this work.

Product Code	DMR100M	
Dimensions	Dry film thickness [μm]	15 ± 1
	Wet film thickness [μm]	19 ± 1
Parameters	Conductivity [mS cm^−1^]	68.6 (H^+^ form)
	Areal density [g m^−2^]	28 ± 1
	Ion exchange capacity [mmol g^−1^]	1.57
Materials	Perfluorinated sulfonic acid polymer, polypropylene reinforcement	

**Table 3:** The charge amount carried by metallic cations removed from different samples in the Donnan dialysis process.

Sample Acid Types	Removed Fe^3+^ [mg]	Removed Al^3+^ [mg]	Removed Mg^2+^ [mg]	Total Charges Removed [mequ.]
WPA	2.79	4.76	21.91	2.48
MPA	4.86	4.27	19.07	2.30
Fe^3+^-SPA	5.60	/	/	0.30
Al^3+^-SPA	/	6.49	/	0.72
Mg^2+^-SPA	/	/	24.04	1.98

**Table 4:** Element contents in the perfluorinated sulfonic acid CEM equilibrated with the synthetic SPA solutions.

Sample Acid Type	P [mg]	Fe^3+^ [mg]	Al^3+^ [mg]	Mg^2+^ [mg]	Target Metal/P [mol/mol]
Fe^3+^-SPA	0.010	0.230	0.010	0.100	13.180
Al^3+^-SPA	0.010	0.001	1.030	0	118.366
Mg^2+^-SPA	0.010	0	0.015	3.091	398.581

## Data Availability

The data presented in this study are openly available in Dryad at DOI https://doi.org/10.5061/dryad.31zcrjdk9, accessed on 9 April 2021.
